# Do hip precautions after posterior-approach total hip arthroplasty affect dislocation rates? A systematic review of 7 studies with 6,900 patients

**DOI:** 10.1080/17453674.2020.1795598

**Published:** 2020-07-28

**Authors:** Jack Crompton, Liza Osagie-Clouard, Akash Patel

**Affiliations:** a Royal Free Hospital, London; b Institute of Orthopaedics and Musculoskeletal Sciences, University College London; c Division of Surgery and Interventional Science, University College London, UK

## Abstract

Background and purpose — Hip precautions limiting flexion, adduction, and internal rotation have been prescribed traditionally to minimize dislocation rates following THA. We assessed the prevalence of hip dislocation following posterior approach total hip arthroplasty without postoperative hip precautions.

Methods — A systematic review of multiple medical databases was performed using the PRISMA guidelines and checklist. All clinical outcome studies that reported dislocation rates and postoperative instructions following posterior approach, primary surgery, published within the last 6 years, were included.

Results — 6,900 patients were included from 7 Level I–IV studies, with 3,517 treated with and 3,383 without precautions. There was no statistically significant difference in the rates of dislocation between groups (2.2% in restricted group vs. 2.0% in unrestricted group). All but 1 study demonstrated no statistically significant differences in patient-reported outcome scores between restricted and unrestricted groups, including those pertaining to return to function, confidence, and pain.

Interpretation — The review found no impact on dislocation rates following total hip arthroplasty performed through a posterior approach, regardless of the use of hip precautions. We also found no impact of the prescription of hip precautions on patient-reported outcome scores.

Prosthesis dislocation is a rare complication of posterior approach THA but with a significant impact on mortality and morbidity. Data suggests 11–24% of revision procedures are secondary to recurrent dislocations with a reported dislocation rate post-THA as high as 2.5%, which was traditionally thought to be influenced by surgical approach (Dargel et al. 2014, Skoogh et al. 2019, van der Weegen et al. 2019). Defunctioned abductors, insufficient capsular or short external rotator repair were purported to lead to an increased risk of dislocation compared with direct anterior surgery. However, multiple large-scale retrospective studies have demonstrated no difference in dislocation rates regardless of approach used (Goldstein et al. 2001, Masonis and Bourne 2002, Chechik et al. 2013, Faldini et al. 2018).

Traditionally, hip precautions were prescribed postoperatively to reduce the risk of dislocation, commonly avoiding hip flexion beyond 90°, adduction beyond the midline, and internal and external rotation greater than 20° (Lucas 2008, Smith and Sackley 2016). With a move towards active recovery plans, earlier unrestricted mobilization is thought to have no impact on dislocation rates. Studies suggest unrestricted mobilization leads to improved functionality, improved clinical outcomes (Khan et al. 2006, Mikkelsen et al. 2014), reduced healthcare costs, and fewer demands on nursing (Coole et al. 2013), despite which hip precautions are still commonly used.

Multiple systematic reviews have investigated the effect of removing hip precautions and the impact on hip dislocation rates. van der Weegen et al. (2016) concluded that reducing hip precautions will not lead to increased dislocation rates post-THA but will improve patient satisfaction. However, Smith et al. (2016) concluded that the evidence at time of publishing was insufficient to determine the potential risks of removing such precautions. Our systematic review focuses on THAs performed using the posterior surgical approach from studies published within the last 6 years and the effect on dislocation rates.

The primary outcome evaluates dislocation rates in patients undergoing posterior-approach THA with postoperative hip precautions reduced or removed. The secondary outcomes are time to dislocation and patient-reported outcome measures, such as Hip Disability and Osteoarthritis Outcome Scores (HOOS), Visual Analogue Scale (VAS) scores, Oxford Hip Scores (OHS), and patient satisfaction rates.

## Methods

The review was conducted in accordance with the PRISMA guidelines. Inclusion criteria for studies were those that assessed posterior approach, demonstrated data regarding use of postoperative instructions, and used patient-reported outcomes. Questionnaires, case studies, and reviews were excluded.

The online databases PubMed, MEDLINE, Web of Science, and the Cochrane Library were searched, with limits between the dates of January 2013 to October 2019. The following search string “hip arthroplasty AND (precautions OR restrictions) AND dislocation” was utilized, including MeSH terms for “arthroplasty, replacement, hip” and “hip dislocation.” From the identified studies, duplicates were removed, and systematic reviews’ bibliographies manually checked for any studies not yet identified in the search.

Abstracts were screened for eligibility, and the eligible studies’ full texts were obtained, where possible, and read to further assess eligibility. Following this, papers were assessed for risk of bias and data extracted from the final studies, using a pre-set data form, including: study year, type of study, number of centers, number of patients, male:female ratio, age, cemented/uncemented, femoral head size, follow-up duration, diagnosis, types of restriction protocol, dislocation rates, time to dislocation, hip outcome scores, pain scores, and time back to ADLs/sport.

### Ethics, funding, and potential conflicts of interest

No ethical approval was required for this study. No funding was received for this work. The authors have no conflicts of interest to declare.

## Results

Through online database searching 202 papers were identified. These were reduced to 112 when the date range was set to January 2013 until October 2019. After removal of duplicates, 66 papers were assessed; of these, 30 were excluded based on their title and abstract. A further 29 papers were excluded based on their full text, leaving the 7 papers used in this study (Mikkelsen et al. 2014, Gromov et al. 2015, Kornuijt et al. 2016, Allen et al. 2018, Dietz et al. 2019, Peters et al. 2019, van der Weegen et al. 2019) (Figure).

**Figure:**
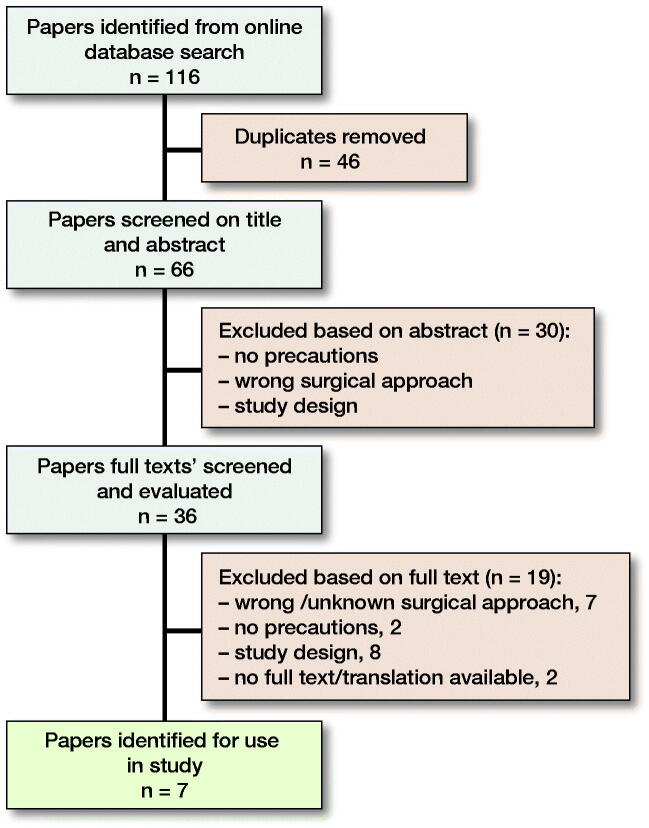
Study flow showing papers searched, screened, and included in the review, with reasons for exclusion.

### Study design and quality

Of the 7 studies included, 5 were prospective and 2 were retrospective, with 2 randomized studies and 5 cohort studies. The follow-up duration ranged from 3 weeks to 1 year, with 2 studies following up on more than 1 occasion. Included were 1 multicenter study from the United States, 3 studies from the Netherlands 2 based in Denmark, and 1 based in the UK (Table 1).

**Table 1.** Data collected from each study using a pre-set data collection form

Authors (study design, number of centers, and follow-up duration)						
	Patients	Male:female	Indication	Prosthesis	Femoral head	Dislocation
Group	n (%)	ratio	for surgery ^a^	fixation, n (%) ^b^	size, n (%)	rate, n/N (%)
Mikkelsen et al. 2014 (Non-randomized controlled study, 1 center, follow-up: 3 and 6 weeks)						
Restricted	146 (40)	53:47	Osteoarthritis	C: 125 (86)	≤ 32 mm: 5 (4)	2/146 (2.7)
				U: 5 (3)	36 mm: 85 (70)	
				H: 15 (10)	40 mm: 29 (24)	
					≥ 44 mm: 3 (2)	
Unrestricted	219 (60)	52:48	Osteoarthritis	C:190 (87)	≤ 32 mm: 9 (4)	6/219 (1.4)
				U: 8 (4)	36 mm: 125 (57)	
				H: 21 (10)	40 mm: 66 (30)	
					≥ 44 mm: 9 (4)	
Allen et al. 2018 (Retrospective cohort study, 1 center, follow-up: 6 weeks)						
Restricted	866 (79.1)	Not	PO: 754	Not reported	Not reported	10/866 (1.15)
		reported	NOF: 7			
			Other ^c^: 105			
Unrestricted	334 (20.9)	Not	PO: 304	Not reported	Not reported	4/334 (1.20)
		reported	NOF: 3			
			Other ^c^: 27			
van der Weegen et al. 2019 (Cohort study, 1 center, follow-up: 90 days)						
Restricted	1,102 (51.2)	37:63	PO: 1,068	C: 597 (54.2)	≤ 28 mm: 594 (53.9)	28/1,102 (2.5)
			Other **^d^**—34	U: 495 (44.9)	≥ 32 mm: 508 (46.1)	
				H: 10 (0.9)		
Unrestricted	1,049 (48.8)	38:62	PO: 1,011	C: 436 (41.6)	≤ 28 mm: 443 (42.2)	17/1,049 (1.6)
			Other **^d^**: 38	U: 597 (56.9)	≥ 32 mm: 606 (57.8)	
				H: 16 (1.5)		
Peters at al. 2019 (Prospective, randomized, non-inferiority study, 1 center, follow-up: 8 week)						
Restricted	203 (49.8)	46:54	Osteoarthritis	Not reported		3/203 (1.48)
Unrestricted	205 (50.2)	40:60	Osteoarthritis	Not reported	32 mm in all patients	3/205 (1.46)
Dietz et al. 2019 (Randomized, controlled study, 3 centers, follow-up: 2–6 weeks, 3–6 months, and 1 year)						
Restricted	145 (51.1)	56:44	NOF excluded	Not reported	35.3 mm **^d^** [34.9–35.7]	2/145 (1.4)
Unrestricted	139 (48.9)	49:51	NOF excluded	Not reported	34.7 mm **^d^** [34–35]	1/139 (0.7)
Kornuijt et al. 2016 (Prospective, comparative safety study, 1 center, follow-up: 3 month)						
Restricted	109 (50.2)	31:69	PO: 101	C: 53 (49)	≤ 28 mm: 51 (47)	1/109 (0.9)
			Other ^d^: 8	U: 56 (51)	≥ 32 mm: 58 (53)	
Unrestricted	108 (49.8)	36:64	PO: 102	C: 40 (37)	≤ 28 mm: 42 (39)	0/108 (0)
			Other **^d^**: 6	U: 68 (63)	≥ 32 mm: 66 (61)	
Gromov et al. 2015 (Retrospective, non-inferiority study, data from DNPR **^e^**, follow-up: 90 days)						
Restricted	946 (41.6)	45:55	Not reported	Not reported	28 mm: 946 (100)	32/946 (3.4)
					32 mm: 0 (0)	
					36 mm: 0 (0)	
Unrestricted	1,329 (58.4)	39:61	Not reported	Not reported	28 mm: 33 (3)	37/1,329 (2.8)
					32 mm: 403 (30)	
					36 mm: 890 (67)	

**^a^**PO = primary osteoarthritis; NOF = neck of femur fracture.

**^b^** C = cemented; U = uncemented; H = hybrid.

**^c^** Secondary arthritis or inflammatory arthritis.

**^d^** Mean (95% CI)

**^e^**DNPR = Danish National Patient Registry.

A risk of bias assessment for our included studies was conducted using “Cochrane’s tool for assessing risk of bias” (Higgins et al. 2011) for the randomized studies, and the “tool to assess risk of bias in cohort studies” by the CLARITY Group (CLARITY Group 2020) to evaluate the cohort studies (Tables 2 and 3, see Supplementary data).

The randomized studies were both at low risk of bias with Dietz et al. (2019) being at high risk for blinding. This was because blinding was not used in this study for either the patients or surgeons. In contrast, Peters et al. (2019) blinded the surgeons to the intervention group to which their patient was allocated.

The cohort studies were at high risk because they were all consecutive studies, comparing the 2 groups at different time points. All of the studies had a low risk with respect to the assessment of the exposure, as surgical records were used in most cases. We can also trust the follow-up for the studies, as any missing data were balanced in both groups and plausible reasons for missing data were stated. 3 studies (Mikkelsen et al. 2014, Kornuijt et al. 2016, Allen et al. 2018) were at a higher risk of bias for matching the exposed and unexposed groups for confounding variables. No statistical adjustment was used in these studies, unlike Gromov et al. (2015), where the odds ratio was adjusted for factors such as age, sex, and femoral head size.

### Patient characteristics

The overall number of patients included in our review was 6,900, with 3,517 allocated to a restricted (or standard precautions) group (RG) and 3,383 allocated to an unrestricted (or reduced precautions) group (UG).

The male:female ratio varied between the different studies; however, 1 study did not record these data (Allen et al. 2018). Overall, there were more females included than males, with 3,375 females compared with 2,325 males. The average age in the studies ranged from 63 to 72 years with details shown in Table 4.

**Table 4.** Average age of patients involved in studies

	Average age	
Study	Restricted	Unrestricted
Mikkelsen et al. 2014, mean (SD)	69 (10)	68 (10)
Allen et al. 2018, mean [IQR]	72 [14]	71 [14]
van der Weegen et al. 2019, mean [IQR]	69 [14]	69 [13]
Peters et al. 2019, mean (SD)	64 (10)	64 (10)
Dietz et al. 2019 mean (95% CI)	63 (61–64)	63 (62–65)
Kornuijt et al. 2016, mean (IQR)	69 [16]	67 [13]
Gromov et al. 2015, mean (range)	67 (20–99)	69 (15–104)

The primary indication for surgery was predominantly osteoarthritis. 1 study (Gromov et al. 2015) did not publish data on the diagnosis while Dietz et al. (2019) specifically excluded patients with neck of femur (NOF) fractures.

### Surgery

5 of the studies (Mikkelsen et al. 2014, Gromov et al. 2015, Kornuijt et al. 2016, Peters et al. 2019, van der Weegen et al. 2019) used the standard posterior approach for their surgeries, whereas Dietz et al. (2019) used the mini-posterior approach. Allen et al. (2018) used a variety of approaches, but we have included only data from the posterior approach surgeries in this study.

The femoral head size used varied between studies, with data on the different diameters used in 4 of the studies (Mikkelsen et al. 2014, Gromov et al. 2015, Kornuijt et al. 2016, van der Weegen et al. 2019). Peters et al. used 32 mm femoral head sized prosthetics on all patients, whilst Dietz et al. (2019) had a mean femoral head diameter of 35.3 mm in the restricted group and 34.7 mm in the unrestricted group. No data were recorded on this in 1 of the studies (Allen et al. 2018). 4 of the studies did not record data on whether the prosthesis was cemented or uncemented (Gromov et al. 2015, Allen et al. 2018, Dietz et al. 2019, Peters et al. 2019), and 1 study reported the use of a constrained liner (Peters et al. 2019).

### Restriction and precaution protocols

3 of the studies used the standard hip precautions, in the restricted groups, of no flexion past 90°, no adduction beyond neutral position and no internal rotation (Mikkelsen et al. 2014, Gromov et al. 2015, Dietz et al. 2019). Mikkelsen et al. and Gromov et al. also gave their patients aids, such as elevated toilet seats, extended shoehorns, sock aids, ergonomic reachers, and bath benches. 2 studies used the same detailed precaution protocol for their restricted groups, including the standard hip precautions as above, with additional restrictions in sleeping, car driving, use of pillows and additional equipment (Kornuijt et al. 2016, van der Weegen et al. 2019). The restricted groups in the study by Allen et al. (2018) seemed to follow the standard hip precautions also, but this was not discussed in detail.

For the unrestricted groups, the majority of studies (Mikkelsen et al. 2014, Gromov et al. 2015, Kornuijt et al. 2016, Allen et al. 2018, Dietz et al. 2019, van der Weegen et al. 2019) enforced few or no precautions or restrictions on patients, with no combined flexion, adduction, and internal rotation being allowed in 3 studies (Mikkelsen et al. 2014, Kornuijt et al. 2016, van der Weegen et al. 2019). Allen et al. (2018) detailed a new protocol for the unrestricted group instructing patients to use movements that were “comfortable” and did not “test their range of movement.” Moreover, Kornuijt et al. (2016) and van der Weegen et al. (2019) advised their patients in the unrestricted group only to drive a car once walking without crutches and to use a pillow for comfort only. Also, in these 2 studies, cross-legged sitting and bending with the operated leg moved backwards was restricted in both groups. Peters et al. (2019) took a different approach by restricting flexion beyond 90°, adduction and rotation past the midline in both groups and only restricting the sleeping position in the restricted group (Table 5, see Supplementary data).

### Dislocation rates

Of the total number of THAs performed (6,900) there were 146 dislocations recorded in the 7 studies, with 78 (2.2%) in the restricted group and 68 (2.0%) in the unrestricted group. Among all the studies, Gromov et al. (2015) had the highest rates of dislocation in both the restricted group (3.4%) and unrestricted group (2.8%), and the lowest hip dislocation rates were from the comparative study by Kornuijt et al. (2016), with 1 (0.9%) dislocation in the restricted group and 0 in the unrestricted group.

Of the 69 dislocations in the study by Gromov et al. (2015), two-thirds of them occurred within 30 days postoperatively, with 63% in the restricted group and 70% in the unrestricted group occurring in the same time period. The 1 dislocation in the Kornuijt et al. (2016) study occurred 2 weeks after the surgery and all of the dislocations reported by Peters et al. (2019) occurred within 3 weeks, with 1 being as soon as 2 days after the operation. The median number of days to dislocation was 4 (IQR 1–15) and 8 (IQR 1–19) in the restricted and unrestricted groups, respectively, reported by van der Weegen et al. (2019). Furthermore, Mikkelsen et al. (2014) reported the number of days after surgery for each dislocation, occurring at 0, 8, 9, 14, 37, and 40 days for the unrestricted group and 13 and 33 days for the restricted group, whilst the time to dislocation was not recorded in 2 studies for the posterior approach (Allen et al. 2018, Dietz et al. 2019).

### Clinical outcomes

3 studies recorded no data on patient-reported outcome (Gromov et al. 2015, Kornuijt et al. 2016, van der Weegen et al. 2019). A form of HOOS was used in 3 studies (Mikkelsen et al. 2014, Dietz et al. 2019, Peters et al. 2019) with Dietz et al. (2019) using the HOOS Jr survey and scores for hip outcomes before and after the intervention. For this study, HOOS Jr scores were taken preoperatively, at 2 weeks, 6 weeks, 3–6 months, and 1 year, with the only significant results being in favor of the restricted group at 2 weeks with a mean score of 68 compared with 64 in the unrestricted group. However, there was no statistically significant difference in scores at any other time point in the study. The authors also used VAS scores to compare groups, with significant improvements at each follow up (p < 0.004), although there were no significant differences between the 2 groups at any stage in the study. There was also no significant difference found in the rate of pain scores given by patients during the study.

Mikkelsen et al. (2014) reported a statistically significant difference in HOOS ADL scores, with the restricted group having the fastest increase in scores; the scores increased by an average of 38 in the restricted group compared with 30 in the unrestricted group in the first 3 weeks postoperatively. There were no significant differences in any of the other HOOS results, including HOOS symptoms, HOOS pain, and HOOS QoL. However, a significantly higher proportion of patients in the unrestricted group were able to perform ADL functions independently 3 weeks postoperatively, including: stair climbing (RG 33%, UG 51%), getting dressed (RG 40%, UG 72%), bath/shower (RG 68%, UG 88%), and house cleaning (RG 38%, UG: 60%).

Patients in both groups reported a significant improvement in function on the HOOS and EQ-5D scores at 8 weeks, according to Peters et al. (2019). The delta scores, calculated by “mean baseline score” minus “mean 8-week score”, were -40 (RG) and -44 (UG) (p = 0.09), for the HOOS scores, and -0.32 (RG) and -0.34 (UG) (p = 0.4) for the EQ-5D scores. VAS scores were also used in this study, again showing no statistically significant differences between the 2 groups (RG 38, UG 40).

Allen et al. (2018) reported skewed results with their satisfaction scores, with all the median scores being 100 (IQR 90–100), likely due to high frequencies of 90 and 100 scores. There was also no significant difference found between the Oxford hip scores (OHS) at baseline or 1 year, with the restricted group’s median score increasing by 21 and the unrestricted group’s median score increasing by 22.

## Discussion

This review found the removal of hip precautions, or reduction of restrictions following a posterior approach THA, did not increase dislocation rates. Using a more restrictive protocol led to an increase in HOOS ADL scores postoperatively, and a statistically non-significant trend for reduced protocols improving ADL scores.

Of the 7 studies included, only 2 were randomized and 5 were single-center studies. A heterogenous group of prosthetics were used in these studies, which could have an impact on the dislocation rates. The differences in precautions between the restricted and unrestricted groups varied between the studies, with most using standard hip precautions in 1 group and few or no precautions in the other group. All but 1 of the studies using vastly different restriction protocols showed lower dislocation rates in the unrestricted group; however, none of these differences reached statistical significance. Our findings of no statistically significant differences in dislocation rates between the protocols was in keeping with reviews of dislocation rates that were not stratified for surgical approach.

A systematic review conducted by van der Weegen et al. (2016) looked at the efficacy of lifestyle restrictions following primary THAs, yet, unlike our study, they included studies conducted as far back as 2005 and covered all types of surgical approach. From the 6 studies they analyzed, no clinically significant negative effect was found when removing or relaxing hip precautions and they also found an improvement in secondary outcomes like our study. Despite this, and other studies (Barnsley et al. 2015, Jobory et al. 2019) reporting similar results with other surgical approaches, hip precautions are still prescribed to patients in as many as two-thirds of hospitals (Gromov et al. 2019).

The variation in whether postoperative restrictions are used for posterior approach THAs might be due to the lack of high-quality evidence, with only 2 randomized controlled studies being found by our searches. Views on how well hip precautions benefit patients also varies, with studies suggesting that some patients may find it difficult to understand or follow the restrictions (Coole et al. 2013), and even that use of precautions may increase anxiety in THR patients (O’Grady et al. 2002). Conversely, some clinicians in the same study by Coole et al. (2013) felt that prescribing hip precautions improved patient confidence on discharge, and aided tissue repair. It would be useful to conduct a multicenter RCT, collecting more detailed data such as patient-recorded outcome measures (PROMs).

Hip dislocation is a multifactorial complication, therefore causality cannot be assessed simply. Various factors are known to affect postoperative risk of dislocation, including age, BMI, sex, comorbidities, surgeon experience, and a host of component factors (Rowan et al. 2018). Moreover, the incidence for hip dislocation is rare and contributes to the difficulty in performing statistically adequate studies with large sample sizes required.

Intraoperative technique can vary and have significant consequences for dislocation rates. A major factor for dislocation risk is the femoral head size of the prosthesis, with hip dislocation incidence increasing with smaller femoral head diameters (Rowan et al. 2018). Therefore, the effectiveness of reducing postoperative restrictions should be interpreted cautiously as no standardized rates were found for posterior-approach THA in our search.

The limitations of our study include the variety of study types included and the lack of some secondary outcomes data, including pain scores, time to return to ADL, time back to work, and functional hip outcome measures. We could only find 2 randomized studies based on our criteria and search. Our included studies had a total of 6,900 patients, suggesting a large sample size; however, a large proportion (64%) of the sample came from only 2 of the studies, one of which used similar restrictions for both groups, only changing sleeping position (Peters et al. 2019).

Ultimately, recent evidence published since 2013 would suggest that removing or relaxing hip precautions and restrictions given to patients following posterior-approach THA has no significant effect on the rates of early dislocation. The paucity of randomized control trials suggests more evidence is needed to determine the significance of effect on return to ADL, work and pain scores, when employing relaxed precautions following posterior approach surgery.

## Supplementary Material

Supplemental Material

## Supplementary data

Tables 2, 3, and 5 are available as supplementary data in the online version of this article, http://dx.doi.org/10.1080/17453674.2020.1795598
