# Supplementary material for: Do hip precautions after posterior-approach total hip arthroplasty affect dislocation rates? A systematic review of 7 studies with 6,900 patients
Source: Acta Orthop. 2020 Jul 28;91(6):687–92. doi: 10.1080/17453674.2020.1795598 (PMC8023879; doi:10.1080/17453674.2020.1795598)
Supplement: Supplemental Material [file IORT_A_1795598_SM0315.pdf]

Supplementary data

Table 2. Risk of bias based on the randomized controlled trials using "The Cochrane Collaboration's tool for assessing risk of bias"

Item	Peters et al. 2019	Dietz et al. 2019
D1: Random sequence generation	High	High
D2: Allocation concealment	Unclear	Unclear
D3: Blinding of participants and personnel	High	Low
D4: Blinding of outcome assessment	Unclear	Low
D5: Incomplete outcome data	High	High
D6: Selective reporting	Low	Low
D7: Other sources of bias	High	High
Overall	High	Unclear

Reference: https://www.ncbi.nlm.nih.gov/books/NBK132494/bin/appf-fm1.pdf (accessed January 25, 2020).

Table 3. Risk of bias based on questions from "Tool to assess risk of bias in cohort studies" for the included cohort studies

Risk of bias question	Mikkelsen et al. 2014	Allen et al 2018	van der Weegen et al.. 2019	Kornuijt et al. 2016	Gromov et al. 2015
Was selection of exposed and non-exposed cohorts drawn from the same population?	Definitely no	Definitely no	Definitely no	Definitely no	Definitely no
Can we be confident in the assessment of exposure?	Definitely yes	Definitely yes	Definitely yes	Definitely yes	Definitely yes
Can we be confident that the outcome of interest was not present at start of study?	Definitely yes	Definitely yes	Definitely yes	Definitely yes	Definitely yes
Did the study match exposed and unexposed for all variables that are associated with the outcome of interest or did the statistical analysis adjust for these prognostic variables?	Definitely no	Definitely no	Probably yes	Probably no	Definitely yes
Can we be confident in the assessment of the p resence or absence of prognostic factors?	Probably yes	Probably yes	Probably yes	Probably yes	Definitely yes
Can we be confident in the assessment of outcome?	Definitely yes	Definitely yes	Definitely yes	Definitely yes	Definitely yes
Was the follow up of cohorts adequate?	Definitely yes	Definitely yes	Definitely yes	Definitely yes	Definitely yes
Were co-Interventions similar between groups?	Definitely yes	Probably yes	Definitely yes	Probably yes	Definitely yes

Reference: https://www.evidencepartners.com/resources/methodological-resources (accessed January 25, 2020).

Table 5. Hip precautions used in each group in the included studies

Study	Restricted group	Unrestricted group
Mikkelsen et al. 2014	No flexion of the hip beyond 90°.<br>No adduction beyond neutral position.<br>No internal rotation.<br>Aids given such as elevated toilet seats, shoehorns, and ergonomic reachers.	No combined full hip flexion, internal rotation and adduction.
Allen et al. 2018	No flexion of the hip beyond 90°.<br>No adduction beyond neutral position.<br>No internal rotatio.n<br>*NB: not well discussed in paper*	
van der Weegen et al. 2019	No flexion of the hip beyond 90°.<br>No adduction beyond neutral position.<br>No internal rotation.<br>Sleep supine for 6 weeks.<br>Pillow at home.<br>Car driving after 6 weeks.<br>Elevated seats (including toilet seat).<br>No cross-legged sitting.	No combined full hip flexion, internal rotation and adduction.<br>No cross-legged sitting.
Peters et al. 2019	No flexion of the hip beyond 90°<br>No adduction beyond neutral position<br>No internal rotation<br>Restricted sleeping position	No flexion of the hip beyond 90°.<br>No adduction beyond neutral position.<br>No internal rotation.<br>No sleeping position restrictions.
Dietz et al. 2019	No flexion of the hip beyond 90°<br>No adduction beyond neutral position<br>No internal rotation	–
Kornuijt et al. 2016	No flexion of the hip beyond 90°<br>No adduction beyond neutral position.<br>No internal rotation.<br>Sleep supine for 6 weeks.<br>Pillow at home.<br>Car driving after 6 weeks.<br>Elevated seats (including toilet seat).<br>No cross-legged sitting.	No combined full hip flexion, internal rotation and adduction.<br>No cross-legged sitting.
Gromov et al. 2015	No flexion of the hip beyond 90°.<br>No adduction beyond neutral position.<br>No internal rotation.<br>Aids given such as elevated toilet seats, shoehorns, and ergonomic reachers.	–
